# Lasers in Esthetic Dentistry: Soft Tissue Photobiomodulation, Hard Tissue Decontamination, and Ceramics Conditioning

**DOI:** 10.1155/2014/927429

**Published:** 2014-07-23

**Authors:** Karen Müller Ramalho, Patrícia Moreira de Freitas, Ana Cecília Correa-Aranha, Marina Stella Bello-Silva, Roberta Marques da Graça Lopes, Carlos de Paula Eduardo

**Affiliations:** ^1^Biodentistry Master Program, School of Dentistry, Ibirapuera University (UNIB), Avenue Interlagos 1329, 04661-100 São Paulo, SP, Brazil; ^2^Special Laboratory of Lasers in Dentistry, Department of Restorative Dentistry, School of Dentistry, University of Sao Paulo, Avenue Professor Lineu Prestes 2227, 05508-000 São Paulo, SP, Brazil; ^3^School of Dentistry, University Nove de Julho (UNINOVE), Rua Vergueiro 44, 01503-001 São Paulo, SP, Brazil

## Abstract

The increasing concern and the search for conservative dental treatments have resulted in the development of several new technologies. Low and high power lasers can be cited as one of these new technologies. Low power lasers act at cellular level leading to pain reduction, modulation of inflammation, and improvement of tissue healing. High power lasers act by increasing temperature and have the potential to promote microbial reduction and ablation of hard and soft tissues. The clinical application of both low and high power lasers requires specific knowledge concerning laser interaction with biological tissues, so that the correct irradiation protocol can be established. The present case report describes the clinical steps of two metal-ceramic crowns development in a 60-year-old patient. Three different laser wavelengths were applied throughout the treatment with different purposes: Nd:YAG laser (1,064 nm) for dentin decontamination, diode (660 nm) for soft tissue biomodulation, and Er:YAG laser (2,940 nm) for inner ceramic surface conditioning. Lasers were successfully applied in the present case report as coadjutant in the treatment. This coadjutant technology can be a potential tool to assist treatment to reach the final success.

## 1. Introduction

In the last few years, the demand for esthetic dentistry has increased significantly. Regarding complex restorations or great alteration in tooth color, indirect composite and ceramic restorations are claimed to be a superior alternative to direct composite resin fillings.

New technologies have been introduced in dentistry and have contributed to the clinical success of restorative procedures. Among these technologies, high and low power lasers have been widely studied and reported as offering many benefits in different steps of restorative treatment [1].

Clinical indications of high power lasers on indirect and direct restorative dentistry include microbial reduction, final caries removal, enamel/dentin etching, internal ceramic conditioning, and crown lengthening. Studies have reported their promising results when used for the treatment/irradiation of the inner surface of ceramic restorations [2–4], as well as for the microbial reduction at the dentin surface and subsurface [5–9]. On the other hand, low power lasers are used for modulation of inflammatory process, pain reduction, and soft tissue biomodulation [10–17].

The present clinical case aims to describe the use of three different wavelengths—low power diode laser (660 nm), Nd:YAG laser (1,064 nm), and Er:YAG laser (2,940 nm)—throughout clinical steps of a ceramic crown placement.

Lasers are contemporary coadjutant tools to conventional dental procedures, which could favor the treatment in order to reach successful final results. This clinical case shows the importance and positive results of using laser technology. Important clinical details and the benefits related to the use of this technology are also addressed.

## 2. Case Presentation

**Figure 1:**
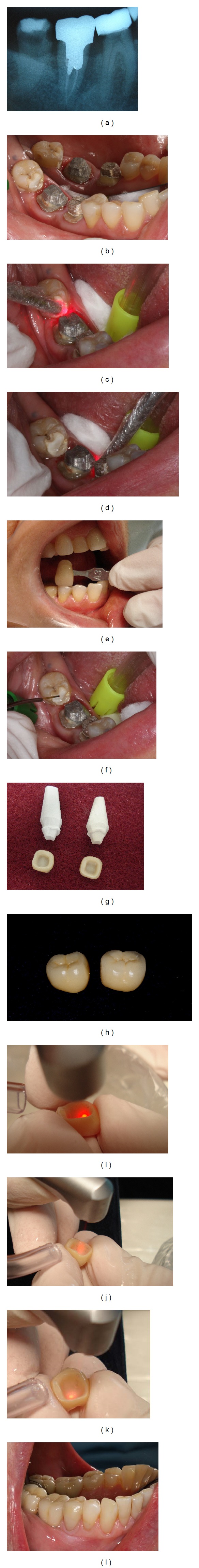
(a) Radiographic exam showing metal-ceramic crowns (teeth 45-46) with unsatisfactory marginal sealing. (b) Metallic posts cementation followed by tooth preparation. (c)-(d) Laser phototherapy (low power laser) applied on periodontal tissues. (e) Selection of tooth color. (f) Microbial reduction using the Nd:YAG laser (1,064 nm). (g)-(h) Porcelain restoration (Zircone LAVA, 3 M/ESPE). (i)–(k) Conditioning of the porcelain inner surface using the Er:YAG laser (2,940 nm). (l) Final luting of the ceramic restoration.

A 60-year-old patient, female, was referred to a private clinic claiming for esthetic restorations of the right inferior molars. The radiographic exam was conducted and revealed two metal-ceramic crowns on both first and second right lower molars with unsatisfactory marginal sealing (Figure 1(a)). The replacement of the inadequate metal-ceramic crowns by indirect ceramic restorations was then indicated.

The patient presented severe teeth grinding and, although metallic crowns seem to be the best indication in this case, patient refused to receive metallic crowns and opted for esthetic ones.

The first clinical step was to remove both crowns and place provisional acrylic-resin crowns. In the following session, endodontic treatment was conducted in the lower right first molar. Following root canals impression of the first molar and the placement of a new metallic post, tooth preparation was conducted, leaving enough space for the ceramic crown (approximately 2 mm from antagonist tooth) (Figure 1(b)). The metallic post of the second molar was maintained.

Instructions of hygiene were given to the patient throughout the prosthetic treatment.

**Figure 2:**
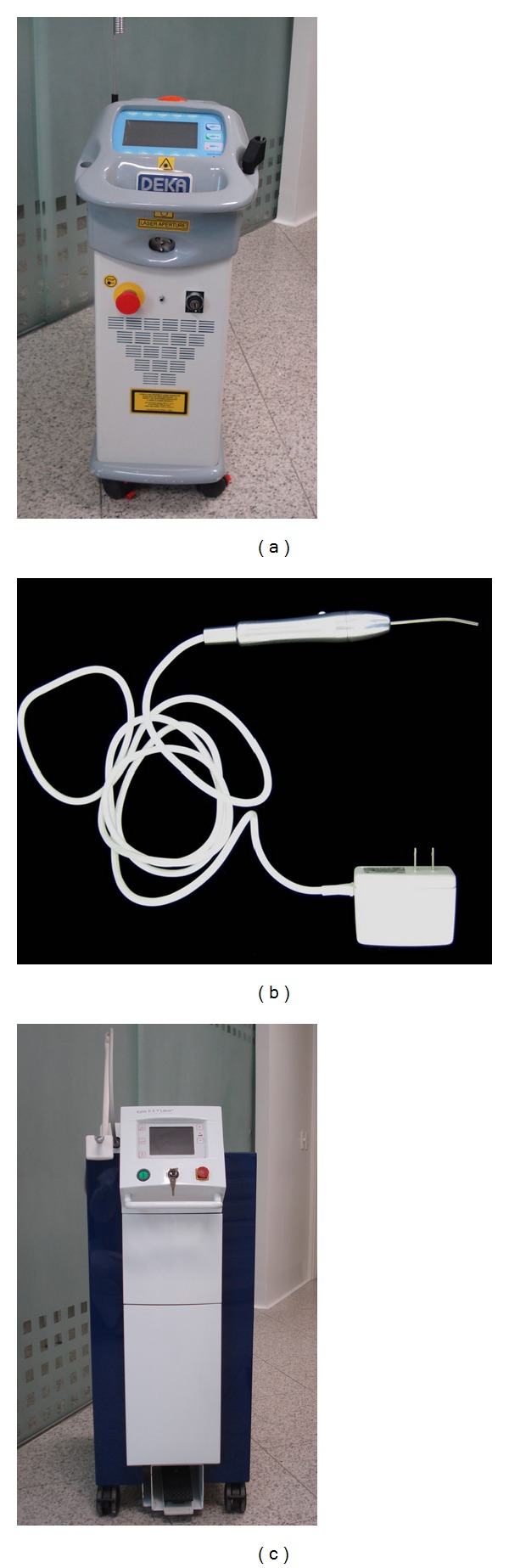
(a) Nd:YAG laser (1,064 nm): microbial reduction; low power laser (660 nm): soft tissue biomodulation; Er:YAG laser (2,940 nm): conditioning of the inner ceramic surface.

After tooth preparation, laser phototherapy with low power laser was performed in all clinical sessions (total of six sessions) on the surrounding periodontal tissue, until the final luting of the ceramic crown was conducted (Figures 1(c), 1(d) and 2(b)). Time interval between laser phototherapy applications varies between two and three days. Laser phototherapy was conducted punctually with a semiconductor laser (InGaAIP—DMC, São Carlos, SP, Brazil), working at 660 nm, using an energy density of 3.57 J/cm^2^ per point and output power of 50 mW. The spot area was 0.028 cm^2^ (Figures 1(c), 1(d)–Figure 2(b)). Each point received a total energy of 0.1 J. A total of 6 points were performed per tooth (2 seconds per point), consisting of 3 points in buccal gingiva and 3 in lingual gingiva. Laser phototherapy with low power laser was carried out aiming to promote soft tissue biomodulation, since both hard and soft tissues should be completely healthy at the end of treatment, so that a fully esthetic outcome could be achieved. Likewise, no inflammatory signals should be present in gingival tissue before final luting procedure.

The ceramic color was selected (Figure 1(e)) and teeth impression was taken with vinyl polysiloxane impression material (Virtual, Ivoclar Vivadent, Schaan, Liechtenstein). In the following session, the ceramic restorations were proved. Prior to the final luting, the decontamination of tooth surfaces was done using a high power laser. The irradiation was performed using the Nd:YAG laser (Smarty A10, DEKA, Firenze, Italy) (Figure 2(a)), 1,064 nm, with the following parameters: 1.5 W, 15 Hz, and 100 mJ. The laser application was made using a 320 *μ*m diameter optical fiber (Figure 1(f)).

Before luting, the temporary restorations were removed and the prepared teeth were cleaned to remove any temporary cement. Both teeth were rinsed with water and air-dried. Next, the shade and fit of the ceramic restorations were checked.

The teeth surfaces and the inner surface of the ceramic restorations were treated and prepared for adhesive luting. Teeth surfaces were conditioned with 35% phosphoric acid (Scotchbond Etchant Phosphoric Acid, 3M ESPE, St. Paul, USA), during 15 seconds, followed by abundant rinse. The inner surfaces of the ceramic restorations (LAVA Zirconia, 3M/ESPE, St. Paul, MN, EUA) (Figures 1(g) and 1(h)) were irradiated with the Er:YAG laser (Key Laser 2, KaVo, Biberach, Germany) (Figure 2(c)) emitting photons at a wavelength of 2.94 *μ*m. The energy per pulse and repetition rate of this equipment range from 60 to 500 mJ and 1 to 15 Hz, respectively. The handpiece number 2052 was used and parameters were set at 250 mJ, 10 Hz, and 2.5 W (Figures 1(i), 1(j), and 1(k)). The irradiation was done under water cooling (5 mL/min), at 12–15 mm distance from the ceramic surface, for 30 seconds (enough time for the entire inner surface to be irradiated). The surfaces were gently dried for 2 seconds.

A thin layer of primer (Multilink Primer A/B—Ivoclar Vivandent, Schaan, Liechtenstein) was applied on the inner surface of the ceramic restoration using a disposable brush and left to react for 3 minutes. Subsequently, it was gently air-dried. The Multilink primer liquids A and B (Ivoclar Vivandent, Schaan, Liechtenstein) were mixed in a 1 : 1 ration and applied with a disposable brush in the entire tooth surface, starting with the enamel, and applied with slight pressure for 15 seconds, according to the manufacturer's instructions. Thirty seconds of reaction time was considered for enamel and 15 seconds for dentin. The applied primer was subsequently air-dried.

The luting cement was dispensed in a double-push syringe, in which the two pastes were mixed in a 1 : 1 ration and then applied on the inner surface of the restorations. The restorations were seated in place and hold (Figure 1(l)). The excesses were immediately removed. The margins of restorations were covered with glycerin gel and the light activation was carried out for polymerization of the luting cement. Finally, the occlusion was checked.

## 3. Discussion

The use of low and high power lasers in dentistry has been, to some extent, adopted as an adjuvant treatment due to its clinical benefits. Successful esthetic treatments involve not only restorative procedures, but also the presence of a healthy surrounding periodontal tissue. After 1971, when Mester [18] first reported the biological effects of low power lasers and the beneficial use of laser phototherapy, this treatment modality has been considered an alternative, noninvasive method to enhance healing of chronic wounds, modulate the inflammatory process, and promote pain relief [16, 19–24]. At the cellular level, the absorption of light by specific chromophore photoreceptors occurs, and once absorbed, the light can modulate cell biochemical reactions and stimulate mitochondrial activity [25]. This primary answer will lead to secondary responses such as increase in ATP synthesis, collagen production, increase in cell proliferation and migration, and biomodulation of inflammatory process [10, 26]. Therefore, the use of laser phototherapy associated with a rigorous routine of hygiene adopted by the patient will lead to a healthy periodontal tissue and contribute not only to a more adequate luting by avoiding inflammatory process and bleeding during impression, but also to a more advanced final esthetic result and longevity of the indirect restoration. Clinical studies have reported laser phototherapy effects on the modulation of periodontal inflammatory process, verifying that patients in whom the conventional treatment was associated with laser phototherapy presented better results than nonirradiated patients [17, 27].

One of the factors that can be related to the success of the treatment provided is the frequency of the laser phototherapy sessions. Several studies have shown that a single irradiation is not as effective as successive ones [28, 29]. Transitory effects in cells resultant from laser phototherapy were also demonstrated in the literature, suggesting that the number of sessions is important to maintain the laser effect on cells [30]. The fluence is also of great importance, since it has been reported that increased effects could be achieved when using laser parameters between 4 and 8 J/cm^2^ [31, 32]. The use of high doses of energy has been considered as a potential inhibitor of cellular proliferation [12]. In the present case report, after teeth preparation, laser phototherapy was applied in gingival tissue surrounding the prepared teeth in all following sessions. Instructions of hygiene were also given to the patient, so that, at the day in which the impression was taken, the gingiva was completely healthy.

The morphology of dentin facilitates accumulation and proliferation of bacteria in dentinal tubules. Sometimes, a provisionary crown does not allow a perfect sealing and dentin contamination can occur. So, it is important that before final luting of a definite crown, dentin decontamination should be performed. The dentin decontamination will be an important step for the success of a prosthetic treatment. There are several studies in the literature that described the potential of Nd:YAG laser in dentin bacteria decontamination [5–9, 33–35]. In general, dental lasers provide greater accessibility of formerly unreachable parts of dentin due to their better penetration into dentinal tissues [35] and a high disinfecting capability of Nd:YAG was reported [5–9, 33–35]. In the present clinical case, the Nd:YAG laser was used before final luting with the aim to promote microbial reduction on dentin substrate. So, the luting of the definitive crown was performed in a decontaminated substrate, aspect that certainly sums to the final success of the treatment.

It is well known that, for adhesive indirect restorations, several steps are required to ensure optimal adhesion of the restoration to the tooth structure and to provide predictable clinical results. These steps include not only the treatment of the dental hard tissues with acid etching and bonding agent, but also the pretreatment of the ceramic inner surface. The mechanical limitations presented by resin composites gave rise to the development of the ceramic systems, which relate esthetics with adequate function and greater resistance to occlusal forces [36]. Zirconia-based ceramics have been increasingly introduced in prosthetic dentistry for the production of fixed partial dentures and crowns in combination with CAD/CAM techniques [37].

Adhesive cementation requires ceramic internal surface treatment, so that micromechanical retentions can be produced and favor adhesion [38]. A further chemical bond is mediated by the silane agent, which favors the union between the ceramic inorganic and the cement organic phases. Because zirconia-based ceramics present an increase in their crystalline content and a decrease in their glass content, conventional surface treatments are not effective enough to produce such alterations. Acid etching and sandblasting have been widely used to promote micromechanical retention in several types of ceramics and are the conventional procedures clinically performed. However, none of these methods are able to produce sufficient surface changes on ceramics for adequate bonding to resin cement [37]. Currently, tribochemical silica-coating (Rocatec System) is considered the most effective method to provide better luting of high crystalline ceramics to dental substrate. This method covers the ceramic surface with a 0.15 *μ*m silica layer and favors its interaction with the silane agent [39]. Recent studies have reported that Rocatec System provides higher bond strength than the conventionally used sandblasting [40–42]. More recently, high power lasers have been investigated for potential use on ceramic conditioning. There are still few studies in the literature about laser treatment of dental ceramics; however, the results indicate that high power lasers can provide similar or even higher bond strength values than those obtained with Rocatec System when adequate irradiation parameters were used [3, 4, 43]. Despite the promising results regarding laser conditioning of high-crystalline ceramics, several studies in the literature do not report this increase in bond strength. For this reason, it is important to emphasize that this improved adhesion to laser conditioned ceramics can only be observed by using adequate laser parameters. Some studies used lower energy or irradiated ceramic surface for a shorter period of time [44, 45]. In this study, the inner surface of the ceramic crowns was irradiated for approximately 30 seconds. The energy of 250 J per pulse and the pulse repetition rate of 10 Hz were used.

As observed in the present clinical case, lasers can be useful for several clinical procedures in dentistry. The association of both low and high power lasers can bring many benefits on biological tissues, improve prosthetic procedures, and provide the patient with a more comfortable and predictable treatment.

## 4. Conclusion

Low and high power lasers can contribute positively to all steps of the indirect restorative treatment period. It is important to highlight that, due to their different interactions with biological tissues, the knowledge on their correct use is of extreme importance for clinical success.
